# Health-related physical fitness and physical activity in elementary school students

**DOI:** 10.1186/s12889-018-5107-4

**Published:** 2018-01-30

**Authors:** Weiyun Chen, Austin Hammond-Bennett, Andrew Hypnar, Steve Mason

**Affiliations:** 1 School of Kinesiology, University of Michigan, 1402 Washington Heights, Ann Arbor, MI 48109 USA; 2 Livonia Public Schools, 15125 Farmington Road, Livonia, MI 48154 USA; 3 Fairfax County Public Schools, Fairfax, VA USA

**Keywords:** Physical fitness, Physical activity, Healthy fitness zone

## Abstract

**Background:**

This study examined associations between students’ physical fitness and physical activity (PA), as well as what specific physical fitness components were more significant correlates to being physically active in different settings for boys and girls.

**Methods:**

A total of 265 fifth-grade students with an average age of 11 voluntarily participated in this study. The students’ physical fitness was assessed using four FitnessGram tests, including Progressive Aerobic Cardiovascular Endurance Run (PACER), curl-up, push-up, and trunk lift tests. The students’ daily PA was assessed in various settings using a daily PA log for 7 days. Data was analyzed with descriptive statistics, univariate analyses, and multiple R-squared liner regression methods.

**Results:**

Performance on the four physical fitness tests was significantly associated with the PA minutes spent in physical education (PE) class and recess for the total sample and for girls, but not for boys. Performance on the four fitness tests was significantly linked to participation in sports/dances outside school and the total weekly PA minutes for the total sample, boys, and girls. Further, boys and girls who were the most physically fit spent significantly more time engaging in sports/dances and had greater total weekly PA than boys and girls who were not physically fit. In addition, the physically fit girls were more physically active in recess than girls who were not physically fit.

**Conclusions:**

Overall, students’ performance on the four physical fitness tests was significantly associated with them being physically active during PE and in recess and engaging in sports/dances, as well as with their total weekly PA minutes, but not with their participation in non-organized physical play outside school.

**Trial registration:**

ClinicalTrials.gov ID: NCT03015337, registered date: 1/09/2017, as “retrospectively registered”

## Background

Currently, almost one-third of U.S. children are overweight or obese [1–4]. To effectively combat the obesity epidemic, the 2008 American Physical Activity (PA) Guidelines recommend that school-aged children and adolescents participate in at least 60 min of moderate-to-vigorous PA each day [5]. However, most children are not engaged in the recommended amount of PA [3, 4]. Cardiovascular fitness is also important because cardiovascular fitness is inversely associated with being overweight in children and adolescents [6–12]. Children with a low fitness level are more likely to become overweight or obese over time than those with a high fitness level [6, 7]. Physical fitness is also a stronger predictor of total and abdominal obesity than PA for children and adolescents [6, 9–12]. As an enabling factor for PA, health-related physical fitness provides the physical foundations for children to enjoy engaging in a variety of physical activities [13, 14]. For example, in a study by Ewrin and Castelli [15], healthy physical fitness was the only significant contributor for elementary school students’ participation in PA out of several factors tested [15]. However, children today are about 15% less fit compared to how fit their parents were when they were young [16]. Therefore, to effectively fight against childhood obesity, there is a critical need for promoting PA participation and increasing physical fitness in school-aged children.

Regular physical activity and health-related physical fitness are key indicators of health outcomes [3, 18, 19]. It has been well documented that regular PA helps maintain a healthy body weight; reduces the risk of developing diabetes, hypertension, and cardiovascular diseases; and improves emotion and stress control [3, 17–19]. Likewise, improving health-related physical fitness, including cardiovascular endurance, muscular strength and endurance, flexibility, and body composition, is conducive to improving health [3, 20–22]. For example, a healthy level of cardiovascular endurance is positively associated with a healthier cardiovascular profile in children [3, 20–22]. Children with a healthy cardiovascular endurance level also have a lower level of overall adiposity and abdominal adiposity [3, 9–12] and low metabolic risk [21]. Hurtig-Wennlöf et al. [22] found that cardiovascular fitness was more strongly related to defining a healthy cardiovascular profile and more negatively associated with cardiovascular disease risk factors compared to objectively measured PA in children. Ortega et al. [3] reported that muscular strength/endurance was also associated with established and emerging cardiovascular disease risk factors. Additionally, improvement in muscular strength/endurance and flexibility had a more positive effect on skeletal health [3, 18] compared to cardiovascular endurance.

Importantly, healthy physical fitness in children and adolescents tends to continue into adulthood [18], and maintaining and enhancing physical fitness is a cornerstone for establishing a physically active lifestyle throughout childhood and adolescence and into adulthood [13, 14, 18]. Studies have shown that children who were physically fit were willing to engage in physical activities and maintain their PA behaviors during their adolescence, whereas children who were physically unfit tended to be physically inactive in adolescence [13–15, 23]. Stodden et al. [13] noted that children who are more physically fit have the foundational physical conditioning required for successful participation in various levels of PA. Therefore, they are more likely to enjoy PA and maintain their interests in PA.

Further, Stodden et al. [13] noted that regular PA and health-related physical fitness are reciprocally associated and enhance one another. Supporting this point, previous studies found that regular PA participation is instrumental to improving and maintaining health-enhancing physical fitness in children [9–12]. Examining the relationship between PA and cardiovascular endurance in children and adolescents, cross-sectional studies have found that greater moderate-to-vigorous PA is significantly and positively associated with a higher level of cardiovascular endurance [9–12, 24–26]. Overall, regular PA habits and healthy physical fitness established in childhood continue into adolescence and adulthood [13, 14, 23].

Given the essential role of PA and health-related physical fitness in promoting and maintaining health, investigating the relationship between PA and physical fitness in children is of great interest and importance. However, previous studies have focused on examining relationships between cardiovascular endurance, body mass index, and PA [9–12, 24]. Despite this, there are other important health-related physical fitness components for school-aged children, including muscular strength and endurance and flexibility [1–4]. Additionally, previous studies have focused on investigating the associations between physical fitness and the overall intensity and amount of PA [9–12, 24–26], rather than examining PA in specific contexts. Thus, there is a paucity of research examining which specific health-related physical fitness components are associated with school-aged children being physically active in a regular physical education (PE) class and recess, as well as being engaged in organized sports/dances and non-organized physical play outside school. It is important to consider these contexts because PE and recess are key settings for providing all children with opportunities to be physically active and to improve fitness during the school day [1, 2, 4]. Likewise, engaging in non-organized physical play and participating in organized sports/dances are major opportunities for children to be physically active and fit outside school [1, 2, 4]. Therefore, there is a critical need for examining what specific fitness components are associated with PA in these various settings.

It has been well documented that girls are less physically active than boys [3, 4, 6, 11]. For example, one study analyzed cross-sectional PA data from the 2003–4 and 2005–6 National Health and Nutrition Examination Survey with youth ages 6–19. The study found that females spent significantly less time in daily moderate-to-vigorous PA compared to males [27]. In another study, 27.9% of adolescent girls were sedentary compared to 10.6% of adolescent boys. Additionally, girls were more likely to play low-to-moderate intensity sports, while boys tended to play high-intensity sports [28]. Given the reciprocal relationship between physical fitness and PA, and given the gender disparity in PA, it is of great importance to investigate what physical fitness components contribute most significantly to boys’ and girls’ PA levels in different PA settings.

Thus, the purpose of this study was twofold: (1) examine associations between four components of physical fitness and PA in four settings, and (2) examine gender differences in the associations between physical fitness components and being physically active in different settings. We hypothesized that: (a) health-related physical fitness components are significantly associated with PA participation in different settings, (b) cardiovascular endurance is more significantly linked to PA participation in most settings compared to other physical fitness components, (c) there are gender differences in associations between physical fitness components and PA in different settings, and (d) children with healthy levels of physical fitness are more physically active than their counterparts with unhealthy levels of physical fitness.

## Methods

### Participants

All fifth-grade students enrolled in eight elementary schools were recruited from the second year of a 3-year *Healthy Kids and Smart Kids* project. The project was designed to help elementary school students become physically active, mentally healthy, and socially cooperative children by implementing an innovative physical education curriculum, a Mileage Club Recess Program, and family and community events. The inclusion criteria for this study were that the fifth-grade students completed all four FitnessGram tests [29], recorded all 7 days of the daily PA log, and returned the parent/guardian-signed consent form. Of 352 fifth-grade students, 349 students returned the signed consent form. Among students who returned the consent form, 84 students were excluded from the study because (a) they incorrectly recorded the time spent in one of four possible settings during weekdays or in one of two possible settings during weekends (e.g., reported more minutes than the maximum minutes [60 min] of PE during a weekday or more minutes than the maximum minutes [30 min] of recess during a weekday); or (b) they did not record minutes spent in PE or recess during weekdays at all, which showed they missed school during that week. A final total of 265 fifth-grade students age 11 years old (133 boys, 132 girls) met the inclusion criteria and comprise the study participants. The university institutional review board approved the project (HUM00088758), and the school district granted permission for us to conduct this study.

### FitnessGram tests

Four FitnessGram tests [29] were used to assess the students’ health-related physical fitness. The FitnessGram test is a validated and reliable health-related fitness assessment toolkit designed by Cooper Institute [29]. “The FitnessGram Standards for Healthy Fitness Zone for Boys” ([29], p. 61) and the “FitnessGram Standards for Healthy Fitness Zone for Girls” ([29], p. 62) were used to determine whether a student’s score on each test was in the Healthy Fitness Zone (HFZ). The HFZ is defined specifically for each test type, age, and gender [30]. The four test items were: (a) a 15-m version of the Progressive Aerobic Cardiovascular Endurance Run (PACER) to assess cardiovascular endurance, (b) a curl-up test to assess abdominal muscular strength and endurance, (c) a push-up test to assess upper body strength and endurance, and (d) a trunk lift test to assess trunk extensor strength and flexibility.

Eight physical education (PE) teachers at the participating elementary schools attended a 3-h FitnessGram test training provided by the first author during one in-service day. The FitnessGram test took place at each elementary school in the last 2 weeks of May. Each PE teacher was asked to follow the testing protocols for administering the four tests to their student in the gymnasium of their school. The testing protocols were: prior to each test, the PE teacher modeled and explained how to perform the test, how to count the number of successful performances, and how to record it using the test recording sheet. Next, the PE teacher organized students into pairs to practice taking the test and recording results. Then, the PE teacher organized the students for taking the test. The PE teacher used one regular PE class to administer the 15-m PACER test and another regular PE class to administer the push-up, curl-up, and trunk lift tests.

#### The 15-m PACER test

For this multistage (21 levels over 21 min) shuttle run, students run from one end across a 15-m area within a lane marked by cones and must touch the other end line by the time a beep sounds. At the sound of the beep, they turn around and run back to the other end. Students continue doing this until they fail to reach the line before the beep two times [30]. Students were instructed to complete as many laps as possible, and the specified pace got faster with each minute that students ran [30]. The score on the PACER test was the number of laps completed successfully. Regarding the HFZ for 15-m PACER test, an 11-year-old boy needs to run between 30 and 94 laps, while an 11-year-old girl needs to run between 19 and 54 laps.

#### The curl-up test

For this test, the student lies in a supine position on a mat with their knees bent at an angle of about 140 degrees, their feet flat on the floor, and their arms straight and parallel to their trunk with the palms of their hands resting on the mat. A 3-in.-wide measuring strip is placed directly underneath their knees on the mat. Keeping their heels in contact with the mat, the student curls up slowly, sliding their fingers across the measuring strip until their fingertips reach the other side (movement distance of 3 in.), and then they curl back down to the starting position. Students were instructed to complete as many curl-ups as possible, up to a maximum of 75. They did this at a specified pace, [30] with the cadence signaled by “up,” “down.” Students were stopped after completing 75 curl-ups, when their second form mistake was made, or when they could no longer continue [30]. Their score was the number of correctly performed curl-ups. The curl-up test HFZ for 11-year-old boys and girls is ≥15 curl-ups.

#### The push-up test

For this test, the student assumes a prone position on the mat with their hands placed under their shoulder; their fingers stretched out; their legs straight, parallel, and slightly apart; and their toes tucked under. The student pushes up off the mat with their arms until the arms are straight, keeping their legs and back straight. The student then lowers their body using their arms until their elbows are bent at a 90 degree angle and their upper arms are parallel to the floor. Students were instructed to perform as many 90° push-ups as possible following a specified cadence signaled by “down,” “up.” The student was stopped when they made the second mistake, such as their knees touching the floor, their upper or lower back swaying, failing to extend their arms fully, or failing to bend to 90 degrees at the elbow. Their score was the number of 90°push-ups they correctly performed. For the push-up test, the HFZ for 11-year-old boys is ≥8 completed push-ups, whereas for girls is ≥7 completed push-ups.

#### The trunk lift test

For this test, the student being tested lies on the mat in a prone position (face down) with their toes pointed and their hands placed under their thighs. The student lifts the upper body off the floor, in a very slow and controlled manner, to a maximum height of 12 in.. A yard stick is placed at least an inch from the front of the student’s chin. Students were instructed to lift their upper body 12 in. off the floor using the muscles of their back and to hold the position long enough to allow for measurement, but they were asked not to exceed 12 in. because excessive arching can cause compression of the discs. Their score was the height the student could hold their chin off the floor. The trunk lift test specifies that lifting the upper body 9 in. off the floor from the prone position as the HFZ cut-off point for 11-year-old boys and girls.

### Physical activity measure

The students’ daily PA minutes were assessed using a daily PA log. After reviewing a validated questionnaire [30] designed to measure children’s 7-day PA and a Fitness ActivityGram [29] designed to measure 3-day PA, the first author designed a 7-day daily PA log for elementary school students in grades 3–5 to record their participation in PA each day. On the 7-day daily PA log, Monday through Sunday are listed in the left column labeled “days.” For each weekday, there are four items in the middle column labeled “where did you do PA,” including PE class, recess, non-organized physical play, and organized sports/dances. For each weekend day, only two items (non-organized play and organized sports/dances) are listed. In the right column labeled “how many minutes”, there are four blank rows for each weekday so participants can record the total minutes spent in PA during a PE class, recess, non-organized physical play, and organized games/dances. In the same column, there are two blank rows for each weekend day for participants to record how many minutes they engaged in non-organized physical play and organized games/dances.

To help students recall what PA they participated in on the previous day and the previous weekend as objectively as possible, each PE teacher worked with students’ home room teachers to monitor the students and ensure they recorded their participation in PA daily. The protocols were: (a) at the beginning of each school day, the students recorded the total minutes of PA they participated in after school through non-organized physical play and organized sports/dance on the previous day or weekend, and (b) at the end of each school day, the students recorded the total minutes of PA they engaged in during PE class and recess on the daily PA log.

The 7-day PA log was a valid and reliable instrument to assess 3rd–5th grade students’ daily PA minutes [31]. In a study [31], we examined students’ self-reported daily PA minutes during the first year (*N* = 1111) and second year (*N* = 1012) as recorded with the 7-day PA log. The results showed that the Cronbach alpha reliability coefficients for PE, recess, non-organized physical play, and organized sports/dances were .94, .79, .78, and .74, respectively, indicating satisfactory internal consistency [32]. In addition, the construct validity of the 7-day PA log was examined by comparing the mean daily PA minutes between year 1 and year 2. The independent samples *t*-test revealed a significant difference in the mean daily PA minutes between the two groups (*t* = 3.04, *df* = 2089, *Sig* (2-tailed) = .002, *p* < .01, *Mean difference* = 5.86). The results indicated that the 7-day PA log could be used to discern the daily PA minutes between the two groups.

### Data analysis

The total weekly PA minutes were computed by summing the total PA minutes spent in PE, recess, non-organized physical play, and organized sports/dances during that week. Then, descriptive statistics were computed for each fitness test and each PA variable, including the PA minutes spent in each activity (PE, recess, non-organized physical play, organized sports/dances) and the total weekly PA minutes. Chi-square analysis was conducted to determine if the percentage of children meeting the HFZ criteria for each fitness test differed for boys and girls, followed by independent samples *t*-tests. Independent samples *t* -tests were also performed to examine if there were mean differences in each PA variable between boys and girls. To determine the relationship between each physical fitness component and each PA variable, multiple R-squared liner regression analyses were performed to examine the relationship between physical fitness components and each PA variable for the total sample, boys, and girls. Subsequently, standardized multiple regression coefficients were analyzed to assess the relative importance of each individual fitness test predicting each PA variable for boys and girls separately. Last, a composite healthy fitness score of the four fitness tests was calculated for boys and girls separately based on the gender-specific cut-off number for reaching the HFZ in each fitness test. Further, an independent samples *t*-test was conducted to examine if there was a significant difference for each PA variable between two levels of physical fitness by gender. All statistical analyses were conducted using IBM SPSS statistics version 24 for windows.

## Results

### Descriptive statistics and Univariate analysis for physical fitness and PA variables

Table 1 presents the descriptive statistics of the four fitness tests for the total sample and by gender. Both boys’ and girls’ mean scores for each fitness test were higher than the cut-off numbers of the HFZs. Boys were most successful at reaching the HFZ for the trunk lift test (92%), followed by the push-up test (81%), curl-up test (80%), and PACER test (56%). Girls were also most successful at reaching the HFZ for the trunk lift test (94%), followed by the curl-up test (83%), push-up test (69%), and PACER test (61%). Chi-square tests revealed a significant difference in the percentages of boys and girls meeting the HFZ for the PACER test (*X*^2^ = 13.27, *df* = 1, *p* < .01) and push-up test (*X*^2^ = 3.89, *df* = 1, *p* < .05). Specifically, significantly more girls met the HFZ for the PACER test compared to boys (based on the gender-specific guidelines). In contrast, significantly more boys met the push-up HFZ criteria than girls (also-based on the gender-specific guidelines). Regardless of the gender guidelines for the HFZ, independent samples *t*-tests revealed that boys statistically outperformed girls in both the PACER test (*t* = 3.73, *df* = 263, *p* < .01, Cohen’s *d* = .46) and push-up test (*t* = 1.98, *df* = 263, *p* < .05, Cohen’s *d* = .24). However, chi-square tests revealed no significant gender differences in percentages of meeting the HFZs in curl-up (*X*^2^ = .05, *df* = 1, *p* > .05) and trunk lift tests (*X*^2^ = .64, *df* = 1, *p* > .05). Likewise, the independent samples *t*-tests showed no significant gender difference in mean scores on the curl-up (*t* = .22, *df* = 263, *p* > .05) and trunk lift tests (*t* = .80, *df* = 263, *p* > .05).

**Table 1:** Descriptive Statistics of Fitness Tests by Gender and Total Sample

	N	Mean ± SD	SE	% of HFZ
PACER				
Boys (HFZ: 30–94 laps)	133	35 ± 17.51	1.52	56%
Girls (HFZ: 19–54 laps)	132	27 ± 16.17	1.41	61%
Total sample	265	31 ± 17.26	1.06	59%
Curl-up				
Boys (HFZ: 15–28)	133	35 ± 22.21	1.93	80%
Girls (HFZ: 15–28)	132	35 ± 21.31	1.86	83%
Total sample	265	35 ± 21.73	1.34	82%
Push-up				
Boys (HFZ: 8–20)	133	16 ± 11.98	1.94	81%
Girls (HFZ: 7–15)	132	13 ± 11.79	1.03	69%
Total sample	265	14 ± 11.95	0.73	75%
Trunk Lift				
Boys (HFZ: 9–12 in.)	133	11.74 ± 3.46	0.30	92%
Girls (HFZ: 9–12 in.)	132	11.47 ± 1.88	0.16	94%
Total sample	265	11.61 ± 2.79	0.17	93%

Table 2 presents descriptive statistics of each weekly PA variable and the total weekly PA minutes by gender. Both boys and girls spent 58 min in PA during PE lessons on average, as the students have two 45-min PE classes per week. Boys spent 96 min and girls spent 88 min in PA during recess through the week. Boys’ mean minutes spent in non-organized physical play were 207, while girls’ were 195. Boys spent 156 min and girls spent 162 min playing organized sports/dances. Independent samples *t*-tests yielded a significant difference in the mean weekly PA minutes in recess between boys and girls (*t* = 2.12, *df* = 263, *p* < .05), but not in PE (*t* = −.34, *df* = 263, *p* > .05), physical play (*t* = .71, *df* = 263, *p* > .05), or sports/dances (*t* = .37, *df* = 263, *p* > .05).

**Table 2:** Descriptive Statistics of Each PA Variable in Minutes and Total PA Minutes by Gender

	Mean ± SD (Boys)	SE	Mean ± SD (Girls)	SE
PE	57.54 ± 5.42	.47	57.77 ± 5.5	.49
Recess	96.03 ± 29.97	2.60	87.84 ± 32.74	2.85
Physical Play	207.73 ± 164.29	14.30	194.57 ± 136.49	11.80
Sports/dances	155.71 ± 191.58	17.13	162.05 ± 227.67	19.81
Total PA	514.73 ± 280.84	24.35	502. 23 ± 269.82	23.48

### Relationship between physical fitness and each PA variable

As seen in Table 3, the results of model 1 indicated that the four physical fitness tests were significantly linked to the weekly PA minutes in PE at *p* < .05, accounting for 4% of the total variance. However, the standardized regression coefficients (*β*) indicated that none of the four fitness tests was individually significant linked to the weekly PA minutes in PE. The results of model 2 revealed that boys’ four physical fitness tests did not significantly associated with their weekly PA minutes in PE, accounting for 4% of the total variance. In contrast, the results of model 3 indicated that the girls’ four physical fitness tests were significantly associated with their weekly PA minutes in PE at *p* < .05, explaining 7% of the total variance. Furthermore, the standardized regression coefficients (*β*) revealed that curl-up test was the significant individual contributor to weekly PA minutes at *p* < .01.

**Table 3:** Results of Four Fitness Tests Linking to Weekly PA minutes in PE

	R	R^2^	F	*p*			
Total Sample							
Model 1	.19	.04	2.48	*.044*	Beta	t	P
PACER					−.08	−1.17	.244
Curl-up					.13	1.97	*.050*
Push-up					.10	1.53	.126
Trunk lift					−.09	−1.52	.130
Boys							
Model 2	.19	.04	1.16	.332			
PACER					−.05	−.48	.633
Curl-up					−.00	−.02	.982
Push-up					.15	1.55	.125
Trunk lift					−.12	−1.33	.186
Girls							
Model 4	.27	.07	2.47	*.048*			
PACER					−.13	−1.31	.190
Curl-up					.27	2.87	*.005*
Push-up					.07	.73	.470
Trunk lift					−.01	−.13	.894

Italic indicates a significant level at *p* < 05

Regarding recess, as presented in Table 4, the results of model 1 revealed that the four physical fitness tests were significantly associated with the weekly PA minutes in recess at *p* < .05, accounting for 4% of the total variance. Subsequently, the standardized regression coefficients (*β*) indicated that PACER performance was the significant individual contributor to weekly PA minutes at *p* < .05. However, the results of model 2 indicated that the boys’ four physical fitness tests were not significantly linked to their weekly PA minutes in recess, explaining 4% of the total variance. In contrast, the results of model 3 indicated that the girls’ four physical fitness tests were significantly associated with their weekly PA minutes in recess at *p* < .01, explaining 12% of the total variance. Furthermore, the standardized regression coefficients (*β*) indicated that PACER performance was the significant individual contributor to weekly PA minutes at *p* < .05.

**Table 4:** Results of Four Fitness Tests Linking to Weekly PA Minutes in Recess

	R	R^2^	F	*p*			
Total Sample							
Model 1	.21	.04	3.01	*.019*	Beta	t	P
PACER					.14	2.01	*.045*
Curl-up					.03	.48	.629
Push-up					.10	1.49	.139
Trunk lift					.04	.61	.541
Boys							
Model 2	.11	.01	.37	.828			
PACER					.00	.04	.966
Curl-up					−.03	−.33	.742
Push-up					.11	1.12	.264
Trunk lift					−0.01	−.09	.926
Girls							
Model 4	.35	.12	4.39	*.002*			
PACER					.20	2.22	*.035*
Curl-up					.14	1.52	.130
Push-up					.08	.91	.366
Trunk lift					.16	1.83	.069

Italic indicates a significant level at *p* < 05

With respect to the non-organized physical play outside of school, Table 5 shows that the four physical fitness tests were not significantly associated with the weekly PA minutes in non-organized physical play (*p* > .05) for the total sample, boys, and girls.

**Table 5:** Results of Four Fitness Tests Linking to Weekly PA Minutes in Non-Organized Physical Play

	R	R^2^	F	*p*			
Total Sample							
Model 1	.18	.03	2.08	.084	Beta	t	P
PACER							
Curl-up							
Push-up							
Trunk lift							
Boys							
Model 2	.23	.06	1.83	.126			
PACER					.19	1.93	*.056*
Curl-up					.11	1.23	.221
Push-up					−.10	−1.10	.273
Trunk lift					.04	.49	.627
Girls							
Model 4	.20	.04	1.27	.286			
PACER					−.06	−.62	.535
Curl-up					.12	1.24	.218
Push-up					−.14	−1.57	.119
Trunk lift					.11	1.23	.222

Italic indicates a significant level at *p* < 05

Regarding PA in sports/dances, as illustrated in Table 6, the four physical fitness tests were significantly associated with the weekly PA minutes in sports/dances at *p* < .01, accounting for 15% of the total variance. Subsequently, the standardized regression coefficients (*β*) indicated that each fitness test was also individually associated with the weekly PA minutes in sports/dances at *p* < .05. Likewise, the results revealed that the boys’ four physical fitness tests were significantly associated with their weekly PA minutes in sports/dances at *p* < .01, accounting for 23% of the total variance. Further, the standardized regression coefficients (*β*) indicated that PACER, curl-up, and push-up performance was individually linked to the weekly PA minutes in sports/dances at *p* < .05, but trunk lift was not. Similarly, the results indicated that the girls’ four physical fitness tests were significantly associated with their weekly PA minutes in sports/dances at *p* < .01, explaining 11% of the total variance. Furthermore, the standardized regression coefficients (*β*) indicated that push-up performance was individually associated with the weekly PA minutes in sports/dances at *p* < .05.

**Table 6:** Results of Four Fitness Tests Linking to Weekly PA Minutes in Sports/Dances

	R	R^2^	F	*p*			
Total Sample							
Model 1	.39	.15	11.35	*.000*	Beta	t	P
PACER					.15	2.36	*.019*
Curl-up					.14	2.36	.019
Push-up					.21	3.33	*.001*
Trunk lift					.12	2.09	*.037*
Boys							
Model 2	.48	.23	9.36	*.000*			
PACER					.23	2.70	*.008*
Curl-up					.19	2.27	*.025*
Push-up					.19	2.26	*.025*
Trunk lift					.13	1.70	.091
Girls							
Model 4	.33	.11	3.83	*.006*			
PACER					.10	1.09	.279
Curl-up					.10	1.12	.266
Push-up					.22	2.44	*.016*
Trunk lift					.11	1.26	.211

Italic indicates a significant level at *p* < 05

For total weekly PA minutes, as seen in Table 7, the results of model 1 revealed that the four physical fitness tests were significantly linked to the total weekly PA minutes at *p* < .01, accounting for 13% of the total variance. The standardized regression coefficients (*β*) indicated that PACER performance was the most significant individual contributor to weekly PA minutes at *p* < .01, followed by curl-up and trunk lift performances at *p* < .05. Push-up performance was not significantly individual contributor to weekly PA minutes. Additionally, the results of model 2 revealed that the boys’ four physical fitness tests were significantly associated with their total weekly PA minutes at *p* < .01, accounting for 19% of the total variance. Subsequently, the standardized regression coefficients (*β*) indicated that PACER performance was the most significantly individual contributor to weekly PA minutes at *p* < .01, followed by curl-up performance at *p* < .05. Push-up and trunk lift performances were not significantly individual contributor to weekly PA minutes. Likewise, the results of model 3 indicated that the girls’ four physical fitness tests were significantly linked to their total weekly PA minutes at *p* < .01, explaining 9% of the total variance. However, the standardized regression coefficients (*β*) indicated that none of the physical fitness test alone was a significantly individual contributor to their weekly PA minutes.

**Table 7:** Results of Four Fitness Tests Linking to Total Weekly PA Minutes

	R	R^2^	F	*p*			
Total Sample							
Model 1	.36	.13	9.7	*.000*	Beta	t	P
PACER					.18	2.82	*.005*
Curl-up					.16	2.70	.009
Push-up					.10	1.62	.115
Trunk lift							
Boys							
Model 2	.43	.19	7.35	*.000*			
PACER					.28	3.10	*.002*
Curl-up					.18	2.14	*.035*
Push-up					.07	0.85	.413
Trunk lift					.12	1.49	.131
Girls							
Model 4	.30	.09	3.19	*.017*			
PACER					.08	0.81	.406
Curl-up					.17	1.82	.082
Push-up					.12	1.35	.186
Trunk lift					.17	1.91	.060

Italic indicates a significant level at *p* < 05

### Differences in PA variables between healthy and unhealthy fitness levels

**Fig. 1:**
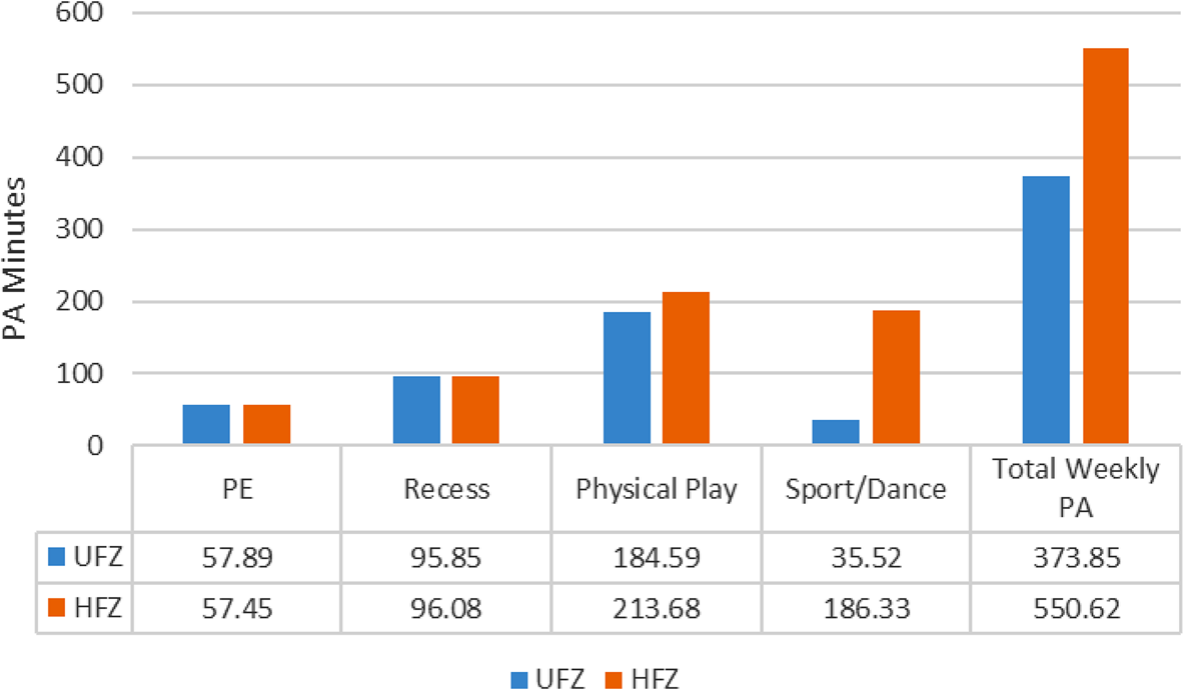
Boys’ mean PA minutes in each PA variable between the two groups

The cut-off number for the composite HFZ score was 62 for boys. Accordingly, 106 boys whose fitness scores were 62 or greater were classified as having healthy fitness levels, while 27 whose fitness scores were lower than 62 had unhealthy fitness levels. Figure 1 illustrates the mean weekly PA minutes in each setting between the healthy and unhealthy fitness groups for boys. The results of independent samples *t*-tests revealed that physically fit boys spent significantly more minutes in sports/dances (*t* = 6.42, *df* = 128.32, *p* < .01) and had greater total weekly PA (*t* = 3.85, *df* = 61.44, *p* < .01) than boys who were unfit. In contrast, there were no significant fitness group differences in mean PA minutes spent in PE (*t* = .37, *df* = 40.52, *p* > .05), recess (*t* = .03, *df* = 36.41, *p* > .05), and non-organized physical play (*t* = .82, *df* = 40.49, *p* > .05) for boys.

**Fig. 2:**
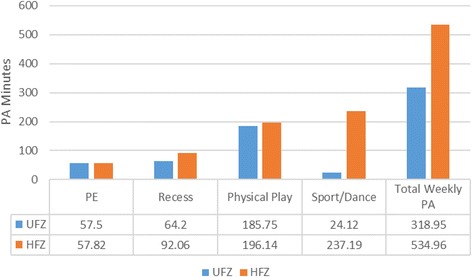
Girls’ mean PA minutes in each PA variable between the two groups

For girls, the cut-off number for the composite HFZ score was 50. Accordingly, 112 girls who scored 50 or greater were classified as having healthy physical fitness levels, while 20 who scored lower than 50 were classified as having unhealthy physical fitness levels. Figure 2 shows the mean weekly PA minutes in each setting between the two groups for girls. The results of independent samples *t*-tests yielded a significant difference between the two groups, where physically fit girls had greater mean weekly PA minutes during recess (*t* = 3.59, *df* = 25.79, *p* < .01), sports/dances (*t* = 7.69, *df* = 46.18, *p* < .01), and total weekly PA (*t* = 5.13, *df* = 46.18, *p* < .01). In contrast, there were no significant fitness group differences in mean PA minutes spent in PE (*t* = .218, *df* = 24.67, *p* > .05) and non-organized physical play (*t* = .313, *df* = 26.03, *p* > .05) for girls.

## Discussion

As expected in hypothesis 1, children’s cardiovascular endurance, muscular strength and endurance, and flexibility determined by the fitness tests were significantly associated with their total weekly PA minutes. These findings are supported by work from Sallis et al. [25] which assessed the health-related physical fitness components of 528 10-year-old children using FitnessGram testing and their current levels of PA using child self-reports. The study [25] found that the school-aged children’s four health-related physical fitness components were significantly related to their levels of physical activity. The unique findings of the present study add to the literature that the four health-related physical fitness components are significantly associated with children’s PA minutes during PE and recess, although explaining only small percentages of the variance in both settings. The children’s physical fitness components were also significantly associated with their PA minutes during organized sports/dances outside school, explaining a relatively large proportion of the total variance. However, the four physical fitness components were not significantly linked to the children’s engaging in non-organized physical play outside of the school.

The results partially supported hypothesis 2. This study indicated that children’s healthy level of cardiovascular endurance was the most significant correlate of their total weekly PA minutes. Similarly, previous studies found a significant association between amount of PA and cardiovascular endurance in school-aged children [9–12, 24, 25]. Cardiovascular endurance was the most significant link to children’s total weekly PA in and outside school in this study. Given that sports play and dance activities are classified into the vigorous intensity level [33], this study supports conclusions made by previous studies [9–11] that a healthy level of cardiovascular endurance is associated with vigorous PA such as competitive and noncompetitive sports and physical activities. However, in this study, only 56% of boys’ and 61% of girls’ cardiovascular endurance was in the healthy fitness range. Compared to the other health-related fitness components, boys and girls in this study exhibited the lowest percentage of meeting the healthy fitness criteria for cardiovascular endurance. This suggests that there is an urgent need for improving boys’ and girls’ cardiovascular endurance.

Also, the present study uniquely found that children’s healthy level of abdominal muscular strength and endurance and of back extensor strength and flexibility were all significant correlates of their total weekly PA minutes, in addition to cardiovascular endurance. For children’s total weekly PA minutes, this study found that children’s healthy levels of abdominal muscular strength and endurance was the only significant correlate of their PA minutes in PE lessons. Children’s healthy levels of upper body strength was the most significant correlate of their engaging in sports and dances outside school. Additionally, children’s healthy levels of abdominal muscular strength and endurance and back extensor strength and flexibility along with PACER test performance all were significant individual correlates of their participating in sports and dances outside school. Upper body and abdominal muscular strength and endurance are the bedrock for successfully performing object control skills used in team sports, individual sports, and lifetime sports [13, 23, 34]. Playing team and individual sports such as basketball, football, baseball, softball, and tennis also require and work on upper and abdominal muscular strength and endurance. Thus, their relationships are reciprocal [13, 23, 34]. In this study, the promising results indicated that most boys’ and girls’ abdominal and upper-body muscular strength and endurance and their back extensor strength and flexibility were in the healthy fitness levels. In conjunction with cardiovascular endurance, these fitness components together were significantly associated with both boys’ and girls’ engaging in PA during school and participating in organized sports/dances outside of school.

As expected in research hypothesis 3, the associations between physical fitness components and PA in various settings were gender specific. The results indicated that physical fitness played a more significant role in participating in total weekly PA minutes for boys (19% of the total variance) than for girls (9% of the total variance). Of the four physical fitness components, cardiovascular endurance and abdominal muscular strength/endurance were significant correlates of boys’ total weekly PA minutes, while upper-body and back extensor strength and endurance were not significant correlates of boys’ total weekly PA minutes. These results are partially supported by a previous study [25] showing that abdominal and upper-body muscular strength and endurance were significantly associated with PA in boys. In contrast, none of the four fitness components acted as a standalone factor that was significantly linked to girls’ total weekly PA minutes. This is inconsistent with the previous study by Sallis et al., [25] which found that girls’ curl-ups was significantly associated with their PA. Furthermore, the physical fitness components were not significantly linked to boys’ PA minutes during PE and recess. On the contrary, the physical fitness components were significantly associated with girls’ PA minutes during PE and recess. Of the four fitness components, abdominal muscular strength and endurance played a significant role for girls to participate in PA during PE, while having healthy cardiovascular endurance played a significant individual role for girls to participate in PA during recess. This study also found that the four fitness components played a significant role for both boys and girls participation in sports/dances outside of school. Furthermore, each fitness component was the significant individual correlate of boys’ participation in sports and dances, with the cardiovascular endurance as the most significant individual correlate. In contrast, only upper-body strength and endurance was the significant individual correlate of girls’ participation in sports and dances outside of school.

Confirming hypothesis 4, this study provided empirical support for previous findings that a healthy level of physical fitness is an enabling factor for elementary school students to participate in PA [9–12, 24, 25]. In this study, both boys and girls who met the healthy criteria level for all four fitness tests spent significantly greater time engaging in total weekly PA compared to physically unfit children. Previous studies indicated that children with higher physical fitness levels were more likely to participate in PA [9–12, 24, 25]. In accordance with the previous findings, this study indicated that the children who were physically fit were most likely to be physically active both in and outside school, while children who were physically unfit were less likely to be physically active overall. Also, this study showed that the boys and the girls in the healthy fitness group were engaged in significantly more sport/dance activities than those who were not physically fit. Consistent with these results, previous studies found that children with healthy cardiovascular endurance were more likely to participate in both competitive and noncompetitive sports [15, 26]. Uniquely, the girls in the healthy fitness group were significantly more likely to be physically active during recess compared to girls with unhealthy fitness levels.

Overall, the unique finding of the present study was that the healthy levels of all four physical fitness components played a significant role for boys and girls to be physically active in PE and during recess, and to engage in PA during organized sports/dances outside school. It has been increasingly advocated that schools provide realistic and appropriate settings for improving health-related physical fitness and prompting PA participation [1–4]. Indeed, more than 95% of youth are enrolled in schools and spend about half their waking hours in schools [1]. One key solution for meeting the urgent need to enhance children’s physical fitness and participation in PA is to implement a Comprehensive School-based PA Program [1, 4, 31]. The goal of a Comprehensive School-based PA Program is to engage students in a variety of physical activities before, during, and after the school day by providing quality PE and school-based PA opportunities. Quality PE is at the heart of the Comprehensive School-based PA Program for promoting physical fitness and active behaviors regardless of children’s abilities [1, 2, 4, 31]. Quality PE is a primary forum for equipping students with knowledge, skills, and fitness to become skillful movers and regular participants in PA [1, 4, 31]. Given the significant role of physical fitness for participating in PA during PE and recess as well as playing sports/dances outside school, this study suggests that PA teachers should use a balanced approach to teach a variety of fitness-enhancing games and physical activities. PE teachers need to engage their students to maximize participation in skill practice, game play, and health-related physical activities by providing developmentally appropriate learning experiences. They also need to reduce class management time and increase the time spent in moderate-to-vigorous PA during a lesson [4, 31]. In addition, PE teachers should proactively work with school staff to create physically active, seasonally-appropriate indoor and outdoor recess programs and active classroom break programs [1, 2, 4]. This Comprehensive School-based PA Program provides students with an effective forum to improve their physical fitness and maintain a healthy fitness level.

This study has four notable limitations. The first limitation of this study was that we did not using an objective PA measure, such as an accelerometer, to assess the students’ PA participation in terms of intensity level. Although the daily PA-log was an appropriate tool for the students to record their PA minutes in each setting, it is possible that students could inflate or deflate their data. Additionally, although sports play and performing dance activities were viewed as being a vigorous intensity level of PA, this study did not directly and objectively measure students’ intensity level of PA in the four settings. Despite the high level of support provided for the students in recording their PA time, it must be recognized that the recorded PA minutes were not directly observed. Thus, it will be important to further confirm the validity of the PA log in future research by comparing it to objective measures of PA (e.g., from an accelerometer). Future research may also combine objective measures with the PA log to measure PA more completely (e.g., obtaining objective data while also recording the settings in which PA occurs). The second limitation of this study was the nature of the cross-sectional research design, which did not examine the changes in the effects of health-related physical fitness on participation in PA over time. Similar to previous studies using cross-sectional research designs (10–13, 25, 26), the causal relationship between physical fitness and PA participation could also not be identified. A future study may use a longitudinal or experimental research design to examine how physical fitness components impact students’ PA participation in different settings over time. The third limitation of this study was that the PE teachers organized and administered the four physical fitness tests using the FitnessGram testing protocols in which students were organized into pairs. One group of students was performing each test, while the other group of students counted and recorded their assigned partner’s testing performance using the standardized recording sheet. Then, they switched roles. Although this administration method was feasible and time-effective, this may increase chances of recording errors. To reduce potential recording errors, future research might require that PE teachers or investigators randomly record a subsample of students’ test performances to confirm the accuracy of recording by students. The last limitation of the study was related to potential confounders such as students’ demographic information (e.g., race/ethnicity, SES of their family, levels of parental education), weather conditions, school PA-related policies and facilities on school yards, environment for promoting PA within the community, and parental support, which can influence school-aged children’s PA participation in and outside school. This study did not control for these potential confounding variables because it did not seek to examine factors that may impact students’ physical fitness and PA participation in and outside school. However, these potential confounding variables should be assessed and controlled for when conducting similar studies in the future. In addition, as the present study showed that the physical fitness components accounted for a very small amount of variance in PA during PE and recess and explained moderate amount of variance in PA during sports/dances and total weekly PA minutes, other potential confounders that may also influence PA participation in the different settings include students’ motor skill competency, motivation and attitudes toward PA, the PE teachers’ quality teaching practices, and social support from peers. Determining the role these factors play along with physical fitness in PA engagement in different settings should be explored in the future studies.

## Conclusions

Overall the four physical fitness components were significantly associated with the students’ engaging in PA during PE and recess, as well as participating in sports/dances outside school, but the fitness components were not significantly related to non-organized physical play outside school. Further, the four physical fitness components were significantly linked to girls’ PA minutes during PE and recess, but not to boys’ PA minutes during PE or recess. However, the four fitness components were significantly associated with both boys’ and girls’ participation in sports/dances outside school and their total weekly PA minutes. The boys and girls with healthy levels of physical fitness spent significantly more time engaging in sports/dances and total weekly PA than those with unhealthy fitness levels. In addition, the girls in the healthy group were more physically active during recess than girls who were unfit. Due to the low percentage of boys and girls meeting overall healthy fitness criteria, there is an urgent need to focus on improving boys’ and girls’ overall physical fitness.

## Abbreviations

HFZ: Healthy Fitness Zone
PA: Physical activity
PACER: Progressive Aerobic Cardiovascular Endurance Run
PE: Physical education
UFZ: Unhealthy Fitness Zone
